# Suppression of the phytoene synthase gene (Eg*crtB*) alters carotenoid content and intracellular structure of *Euglena gracilis*

**DOI:** 10.1186/s12870-017-1066-7

**Published:** 2017-07-17

**Authors:** Shota Kato, Mika Soshino, Shinichi Takaichi, Takahiro Ishikawa, Noriko Nagata, Masashi Asahina, Tomoko Shinomura

**Affiliations:** 1 Department of Biosciences, School of Science and Engineering, Teikyo University, 1-1 Toyosatodai, Utsunomiya, Tochigi 320-8551 Japan; 2 Plant Molecular and Cellular Biology Laboratory, Graduate School of Science and Engineering, Teikyo University, 1-1 Toyosatodai, Utsunomiya, Tochigi 320-8551 Japan; 3 Department of Biology, Nippon Medical School, 1-7-1 Kyonan-cho, Musashino, Tokyo 180-0023 Japan; 4 Department of Life Science and Biotechnology, Faculty of Life and Environmental Science, Shimane University, 1060 Nishikawatsu, Matsue, Shimane 690-8504 Japan; 5 Faculty of Science, Japan Women’s University, Bunkyo-ku, Tokyo, 112-8681 Japan; 6 Plant Molecular and Cellular Biology Laboratory, Department of Biosciences, School of Science and Engineering, Teikyo University, 1-1 Toyosatodai, Utsunomiya, Tochigi 320-8551 Japan; 7 Department of Molecular Microbiology, Faculty of Life Sciences, Tokyo University of Agriculture, 1-1-1 Sakuragaoka, Setagaya, Tokyo 156-8502 Japan

**Keywords:** *Euglena gracilis*, Light-induced stress, Carotenoid, Phytoene synthase, *crtB*, Thylakoid, HPLC, Transmission electron microscopy, RNA interference, Double-stranded RNA

## Abstract

**Background:**

Photosynthetic organisms utilize carotenoids for photoprotection as well as light harvesting. Our previous study revealed that high-intensity light increases the expression of the gene for phytoene synthase (Eg*crtB*) in *Euglena gracilis* (a unicellular phytoflagellate), the encoded enzyme catalyzes the first committed step of the carotenoid biosynthesis pathway. To examine carotenoid synthesis of *E. gracilis* in response to light stress, we analyzed carotenoid species and content in cells grown under various light intensities. In addition, we investigated the effect of suppressing Eg*crtB* with RNA interference (RNAi) on growth and carotenoid content.

**Results:**

After cultivation for 7 days under continuous light at 920 μmol m^−2^ s^−1^, β-carotene, diadinoxanthin (Ddx), and diatoxanthin (Dtx) content in cells was significantly increased compared with standard light intensity (55 μmol m^−2^ s^−1^). The high-intensity light (920 μmol m^−2^ s^−1^) increased the pool size of diadinoxanthin cycle pigments (i.e., Ddx + Dtx) by 1.2-fold and the Dtx/Ddx ratio from 0.05 (control) to 0.09. In contrast, the higher-intensity light treatment caused a 58% decrease in chlorophyll (*a* + *b*) content and diminished the number of thylakoid membranes in chloroplasts by approximately half compared with control cells, suggesting that the high-intensity light-induced accumulation of carotenoids is associated with an increase in both the number and size of lipid globules in chloroplasts and the cytoplasm. Transient suppression of Eg*crtB* in this alga by RNAi resulted in significant decreases in cell number, chlorophyll, and total major carotenoid content by 82, 82 and 86%, respectively, relative to non-electroporated cells. Furthermore, suppression of Eg*crtB* decreased the number of chloroplasts and thylakoid membranes and increased the Dtx/Ddx ratio by 1.6-fold under continuous illumination even at the standard light intensity, indicating that blocking carotenoid synthesis increased the susceptibility of cells to light stress.

**Conclusions:**

Our results indicate that suppression of Eg*crtB* causes a significant decrease in carotenoid and chlorophyll content in *E. gracilis* accompanied by changes in intracellular structures, suggesting that Dtx (de-epoxidized form of diadinoxanthin cycle pigments) contributes to photoprotection of this alga during the long-term acclimation to light-induced stress.

**Electronic supplementary material:**

The online version of this article (doi:10.1186/s12870-017-1066-7) contains supplementary material, which is available to authorized users.

## Background

*Euglena gracilis* is a microalga that has attracted much attention as a potential feedstock for biodiesel production. In outdoor cultivation for biofuel production, direct sunlight of high intensity can cause photoinhibition in microalgae and decrease the algal cell productivity [1, 2]. In photosynthesis of oxygenic phototrophs, excess light energy can generate various reactive oxygen species (ROS), such as superoxide radical (O_2_^−^), hydrogen peroxide (H_2_O_2_), and hydroxyl radical (·OH) in the electron transport chain [3, 4] and singlet oxygen (^1^O_2_^*^) in antenna complexes [5, 6]. ROS (such as ^1^O_2_^*^ and H_2_O_2_) have been shown to cause the cleavage of D1 protein in photosystem II (PSII) in vitro [7–9]. In addition, several studies [10, 11] have shown that ROS inhibit the repair of photodamaged PSII in vivo. When the reaction rate of photodamage to PSII exceeds the rate of repair, photoinhibition of photosynthesis occurs. To minimize this photoinhibition, plants have evolved several protective mechanisms such as chloroplast movement, screening of radiation, ROS scavenging, thermal energy dissipation, cyclic electron flow, and photorespiration [12].

In addition to their light-harvesting function, carotenoids contribute to photoprotection. They dissipate excess excitation energy of singlet-state chlorophylls as heat in xanthophyll-dependent non-photochemical quenching in oxygenic phototrophs [13]. Carotenoids also quench triplet-state chlorophylls in the antenna complex and singlet oxygen in the reaction center of PSII [6, 14, 15]. In general, PSII contains β-carotene in reaction center complexes [16, 17]. Lutein, 9′-*cis* neoxanthin and xanthophyll cycle pigments (violaxanthin and zeaxanthin) are components of antenna complexes of PSII [18, 19].

More than 750 structurally defined carotenoids have been identified in various photosynthetic and non-photosynthetic organisms including bacteria, archaea, fungi, algae, land plants, and animals [20]. Algae have evolved diverse pathways for carotenoid biosynthesis, and some algae synthesize division/class-specific carotenoids; e.g., the allenic carotenoids fucoxanthin in brown algae and diatoms, 19′-acyloxyfucoxanthin in Haptophyta and Dinophyta, and peridinin in dinoflagellates and the acetylenic carotenoids alloxanthin, crocoxanthin and monadoxanthin in Cryptophyta, and diadinoxanthin (Ddx) and diatoxanthin (Dtx) in Heterokontophyta, Haptophyta, Dinophyta and Euglenophyta [21]. The order Euglenida, which includes *E. gracilis,* synthesizes β-carotene and xanthophylls such as zeaxanthin, 9′-*cis* neoxanthin, Ddx, and Dtx [21–24].

Phytoene synthesis, the first step of carotenoid biosynthesis, by phytoene synthase (CrtB, also called Psy) is one of the rate-limiting steps in carotenoid biosynthesis [21, 25]. Steinbrenner and Linden [26, 27] reported that the expression of the phytoene synthase gene (*psy*) in *Haematococcus pluvialis* is induced in response to increased illumination. In addition, several studies have demonstrated light-induced accumulation of carotenoids in certain green algae, such as *H. pluvialis* [26, 27], *Dunaliella salina* [28, 29], and *Chlorella zofingiensis* [30, 31]. Consistent with these reports, our previous studies [32] revealed that high-intensity light (continuous illumination at 920 μmol m^−2^ s^−1^) increased the expression of the phytoene synthase gene in *E. gracilis* (Eg*crtB*), and this finding suggested that high-intensity light induces the accumulation of pigments assumed to be carotenoids in this alga.

To elucidate changes in carotenoid accumulation in *E. gracilis* in response to light stress, we analyzed the content and molecular species of carotenoids in cells grown under various light intensities. We found that the total carotenoid content in *E. gracilis* cells increased in response to light-induced stress. In particular, we found that light-induced stress resulted in an increase in the pool size of diadinoxanthin cycle pigments (Ddx and Dtx) and caused changes in intracellular structures, including chloroplasts. In addition, we transiently silenced Eg*crtB* expression using RNA interference (RNAi) in *E. gracilis* cells and found that the suppression of Eg*crtB* markedly decreased the proliferation and chlorophyll and carotenoid content accompanied by changes in intracellular structures under continuous illumination, even at a standard light intensity. Furthermore, we found that the Dtx/Ddx ratio was significantly increased by both light-induced stress and suppression of Eg*crtB*, suggesting that Dtx (de-epoxidized form of diadinoxanthin cycle pigment) contributes to photoprotection of *E. gracilis* during the long-term acclimation to light-induced stress.

## Results

### Effects of high-intensity light on the content of chlorophyll *a* and *b* in *E. gracilis* cells

**Fig. 1:**
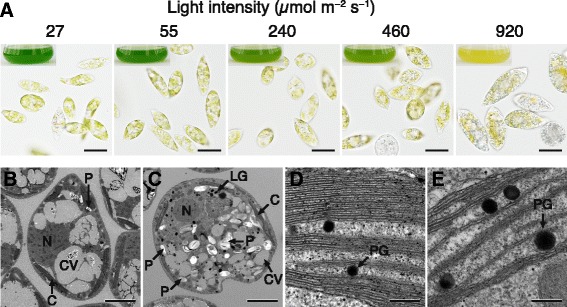
Effects of light intensity on the physical appearance of *E. gracilis* cells. **a** Algal cells and appearance of culture medium (*insets*) after cultivation for 7 days at 25 °C under continuous light at the indicated intensities. Scale bar, 20 μm. **b** and **c** Internal structure of cells grown under illumination at 55 (**b**) or 920 μmol m^−2^ s^−1^ (**c**) for 7 days. Scale bar, 5 μm. **d** and **e** Sections of chloroplasts of cells illuminated at 55 (**d**) or 920 μmol m^−2^ s^−1^ (**e**). Scale bar, 200 nm. C, chloroplast; CV, contractile vacuole; LG, lipid globule; N, nucleus; P, paramylon; PG, plastoglobule

*E. gracilis* cells were grown under continuous illumination in a range of 27–920 μmol m^−2^ s^−1^ for 7 days (Fig. 1a). Growth under 240 μmol m^−2^ s^−1^ yielded cells that looked pale green compared with control cells illuminated at a standard light intensity (55 μmol m^−2^ s^−1^). Indeed chlorophyll *a* and *b* content in these cells was 69% and 70%, respectively, of control cells, although the cell concentration did not differ significantly from control cells (Table 1). Similarly, after cultivation for 7 days under 460 μmol m^−2^ s^−1^, the cellular chlorophyll *a* and *b* content decreased to 61% and 59%, respectively, of control cells, whereas cell concentration increased as much as the control (Table 1). Cultivation under continuous light at 920 μmol m^−2^ s^−1^ for 7 days significantly decreased the cell concentration by 75% compared with control cells; moreover, this high-intensity light decreased chlorophyll *a* and *b* content by 58% and 55%, respectively, relative to the control.

**Table 1:** Effect of high-intensity light on the growth and chlorophyll content of *E. gracilis*

Treatment (μmol m^−2^ s^−1^)	Final cell concentration (×10^6^ cells ml^−1^)	Cell weight (mg FW 10^6^ cells^−1^)	Chlorophyll content (nmol 10^6^ cells^−1^)		Chlorophyll *a*/*b*
			*a*	*b*	
27	1.9 ± 0.1^a^	2.2 ± 0.2^a^	9.2 ± 0.8^a^	1.3 ± 0.3^a^	7.1 ± 0.7^a^
55	1.9 ± 0.1^a^	2.6 ± 0.1^a^	8.9 ± 0.4^a^	1.3 ± 0.1^a^	6.9 ± 0.3^a^
240	2.0 ± 0.0^a^	3.0 ± 0.1^a^	6.1 ± 0.1^b^	0.9 ± 0.0^b^	6.7 ± 0.2^a^
460	2.0 ± 0.0^a^	2.7 ± 0.1^a^	5.4 ± 0.3^b^	0.8 ± 0.1^b^	7.0 ± 0.2^a^
920	0.5 ± 0.1^b^	5.5 ± 0.6^b^	3.7 ± 0.3^c^	0.6 ± 0.1^b^	6.5 ± 0.7^a^

Data represent the mean ± SD of biological triplicates. Different letters in each column indicate a significant differences (*P* < 0.05, Tukey’s test)

Cultivation for 7 days under 920 μmol m^−2^ s^−1^ yielded cells that appeared much larger than those illuminated at the standard light intensity, and the fresh weight of the cells was twice that of the control cells (Fig. 1a and Table 1). Furthermore, in contrast to cells grown under other light intensities, these cells appeared yellow-orange or reddish-orange and accumulated greater numbers of grayish granules thought to be composed of paramylon (~1–2 μm in diameter) in the cells.

### Ultrastructure of *E. gracilis* cells grown under high intensity light

Figure 1c and e show the internal structure of cells and chloroplasts of *E. gracilis* grown under illumination at 920 μmol m^−2^ s^−1^; transmission electron microscopy (TEM) revealed a decrease in the number of thylakoid membranes in chloroplasts by approximately half compared with control cells grown under standard light intensity (Fig. 1b and d). TEM also revealed that the algal cells grown under the high-intensity light contained more plastoglobules (lipid globules in the interthylakoid space of chloroplasts) than control cells and that the plastoglobules of those cells were obviously larger than those in the control (Fig. 1d and e). The high-intensity light also markedly increased the number of osmium-philic droplets (lipid globules) in the cytoplasm compared with control (Fig. 1c).

### Effects of high-intensity light on the relative content of carotenoids in *E. gracilis* cells

**Fig. 2:**
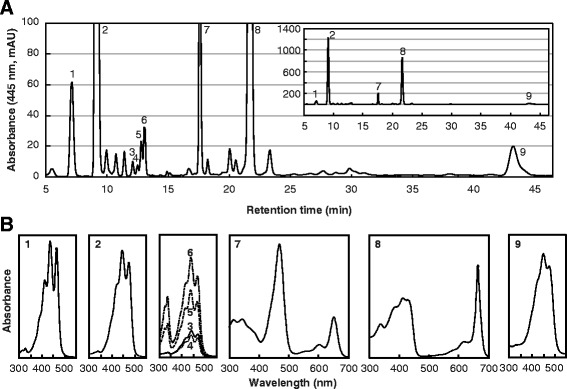
Analysis of carotenoid species in *E. gracilis* with HPLC. **a** HPLC chromatogram (445 nm) of extracts from *E. gracilis*. (*Inset*) The same chromatogram with an expanded *y* axis. mAU, milli-absorbance units. **b** Absorbance spectrum of individual peaks of major carotenoids (peaks 1–6 and 9). 1, neoxanthin; 2, diadinoxanthin; 3, all *trans*-diatoxanthin; 4–6, *cis*-diatoxanthin; 7, chlorophyll *b*; 8, chlorophyll *a*; 9, β-carotene

**Fig. 3:**
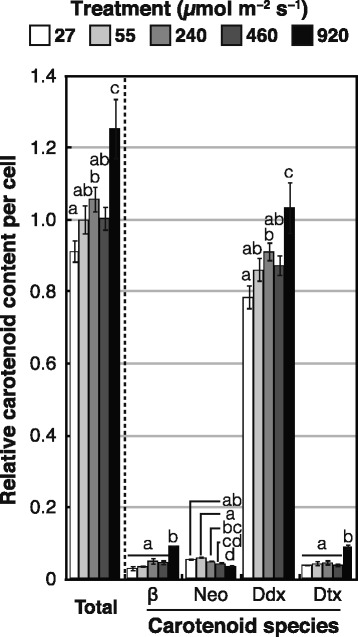
Effects of light intensity on carotenoid content of *E. gracilis* cells. Cells were grown under the indicated light intensities for 7 days. Relative carotenoid content per cell was calculated by normalizing molar ratios of major carotenoid species to chlorophyll *a* content. β, β-carotene; Neo, neoxanthin; Ddx, diadinoxanthin; Dtx, diatoxanthin. Levels of carotenoids per cell are expressed relative to total carotenoids in the cells illuminated at the standard light intensity of 55 μmol m^−2^ s^−1^. Error bars indicate ± SD of biological triplicates. Bars labeled with the same letter are not significantly different (Tukey’s multiple range test, *P* < 0.05)

To identify carotenoid species in *E. gracilis*, we subjected cell extracts to high-performance liquid chromatography (HPLC) and measured absorption of the effluent at 445 nm (Fig. 2a). For control cells grown under illumination with 55 μmol m^−2^ s^−1^, HPLC analyses indicated that β-carotene, neoxanthin, Ddx and Dtx were the major carotenoids and accounted for 4, 6, 86, and 4%, respectively, of the total carotenoids (Fig. 2b). These four carotenoids were also the major species in cells grown under light of higher intensities (Additional file 1), and Fig. 3 shows the relative content of the major carotenoids in those cells. For cells illuminated at 240, 460, or 920 μmol m^−2^ s^−1^, neoxanthin content per cell significantly decreased by 19, 28, and 40%, respectively, relative to control cells; illumination with 27, 240, or 460 μmol m^−2^ s^−1^ had no obvious effect on the content of β-carotene, Ddx and Dtx relative to the control. In contrast, illumination at 920 μmol m^−2^ s^−1^ substantially increased the β-carotene, Ddx and Dtx content per cell by 2.6, 1.2, and 2.1-fold, respectively, compared with control cells, and the total major carotenoids per cell increased by 25% (Fig. 3).

### Suppression of Eg*crtB* expression

**Fig. 4:**
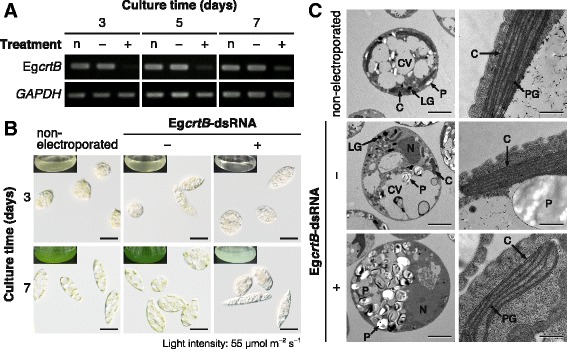
Effects of suppressing Eg*crtB* on *E. gracilis* cells. Cells treated with or without Eg*crtB*-dsRNA were cultured for 7 days at 25 °C under continuous illumination at 55 μmol m^−2^ s^−1^. **a** Semi-quantitative reverse transcription-PCR analysis of Eg*crtB* expression in cells with (+) or without (−) Eg*crtB*-dsRNA. Non-electroporated cells (n) were used as the control. *GAPDH* expression was used as an internal control for the semi-quantitative RT-PCR analysis. **b** Cells treated with or without Eg*crtB*-dsRNA and visual appearance of culture medium (*insets*) after cultivation for 3 and 7 days. Scale bar, 20 μm. **c** TEM images of intracellular structures (left column: scale bar, 5 μm) and sections of chloroplasts (right column: scale bar, 500 nm) of cells treated with or without Eg*crtB*-dsRNA. C, chloroplast; CV, contractile vacuole; LG, lipid globule; N, nucleus; P, paramylon; PG, plastoglobule

Eg*crtB* expression was suppressed using RNAi mediated by double-stranded RNA (dsRNA). Figure 4a shows expression levels of Eg*crtB* in *E. gracilis* cells treated with dsRNA directed toward a partial sequence of Eg*crtB*. Treatment without Eg*crtB*-dsRNA (electroporation alone) had no obvious effect on Eg*crtB* expression. In contrast, expression of Eg*crtB* in cells cultured for 3 days was markedly decreased by the Eg*crtB*-dsRNA treatment. Although Eg*crtB* expression in Eg*crtB*-dsRNA-treated cells gradually recovered during the full 7-day cultivation period, expression was lower than that in non-electroporated cells. These results indicated that Eg*crtB* expression could be transiently suppressed by treating cells with Eg*crtB*-dsRNA.

When control cells were grown under continuous light (55 μmol m^−2^ s^−1^) at 25 °C for 3 days, treatment with Eg*crtB*-dsRNA decreased the cell concentration to 43% and 61% compared with cells treated without electroporation or Eg*crtB*-dsRNA, respectively (Table 2). Electroporation alone decreased the cell concentration by 29%, but Eg*crtB*-dsRNA-mediated Eg*crtB* suppression caused a further marked decrease in cell concentration after cultivation for 3 days. After cultivation for 7 days, the number of cells treated without Eg*crtB*-dsRNA had increased as much as the non-electroporated cells, whereas the concentration of cells treated with Eg*crtB*-dsRNA had decreased by ~82%.

**Table 2:** Effects of suppressing Eg*crtB* on cell concentration and chlorophyll content of *E. gracilis*

Treatment	Cell concentration		Chlorophyll content (nmol 10^6^ cells^−1^)		Chlorophyll *a*/*b*
	Cultured for 3 days (×10^4^ cells ml^−1^)	Cultured for 7 days (×10^6^ cells ml^−1^)	*a*	*b*	
non-electroporated	7.7 ± 0.7^a^	1.9 ± 0.0^a^	7.7 ± 0.9^a^	1.1 ± 0.1^a^	6.9 ± 0.3^a^
Eg*crtB*-dsRNA(−)	5.5 ± 0.7^b^	1.8 ± 0.1^a^	8.5 ± 1.6^a^	1.2 ± 0.2^a^	7.3 ± 0.3^a^
Eg*crtB*-dsRNA(+)	3.3 ± 0.2^c^	0.3 ± 0.0^b^	1.3 ± 0.1^b^	0.2 ± 0.0^b^	6.1 ± 0.5^b^

Data represent mean ± SD of biological replicates. The number of biological replicates was as follows: cell concentration, *n* = 3; chlorophyll content and chlorophyll *a*/*b* ratio, *n* = 4. Different letters in each column indicate a significant differences (*P* < 0.05, Tukey’s test)

Electroporation alone had no obvious effect on cell appearance (Fig. 4b) or chlorophyll *a* and *b* content (Table 2) compared with non-electroporated cells. In contrast, treatment with Eg*crtB*-dsRNA caused chlorosis in cells after cultivation for 3 days. After cultivation for 7 days, chloroplasts in these cells were still pale green, and the culture medium was mostly clear; moreover, the content of chlorophyll *a* and *b* in Eg*crtB*-suppressed cells was decreased to 17% and 20% of non-electroporated cells, respectively (Table 2).

TEM clearly revealed that Eg*crtB*-suppressed cells accumulated many more cytoplasmic paramylon granules compared with cells treated without electroporation or Eg*crtB*-dsRNA (Fig. 4c, left column). In contrast, Eg*crtB*-suppressed cells contained considerably fewer chloroplasts. When we examined 120–150 sections of individual cells, chloroplasts were found in <5% of sections of Eg*crtB*-suppressed cells, whereas almost all sections of cells treated without electroporation or Eg*crtB*-dsRNA contained several chloroplasts (data not shown). The number of thylakoid layers in Eg*crtB*-suppressed cells was slightly lower than in cells treated without electroporation or Eg*crtB*-dsRNA (Fig. 4c, right column).

**Fig. 5:**
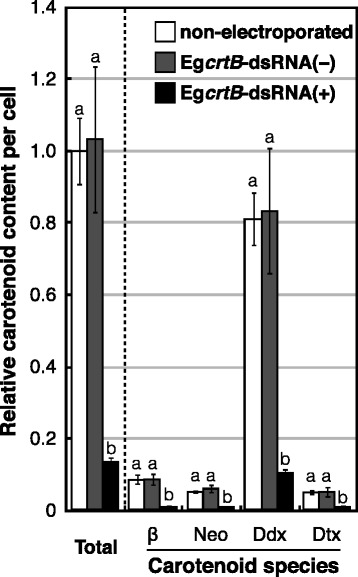
Effects of suppressing Eg*crtB* on carotenoid content of *E. gracilis* cells. Cells were treated with (+) or without (−) Eg*crtB*-dsRNA and cultured for 7 days at 25 °C under continuous light at 55 μmol m^−2^ s^−1^. Non-electroporated cells (n) were used as the control. Levels of carotenoids per cell are expressed relative to total carotenoids in the non-electroporated cells. β, β-carotene; Neo, neoxanthin; Ddx, diadinoxanthin; Dtx, diatoxanthin. Error bars indicate ± SD of biological quadruplicates. Bars labeled with the same letter are not significantly different (Tukey’s multiple range test, *P* < 0.05)

Figure 5 shows the relative content of major carotenoid species in cells treated with or without Eg*crtB*-dsRNA. Treatment without Eg*crtB*-dsRNA had no significant effect on the content of the four major carotenoids in cells cultivated for 7 days. In contrast, treatment with Eg*crtB*-dsRNA drastically decreased the content of the total major carotenoids per cell by 86% relative to non-electroporated cells (Fig. 5). After cultivation for 7 days, the relative content of the four major carotenoids, namely β-carotene, neoxanthin, Ddx and Dtx, in the Eg*crtB*-suppressed cells was 12, 19, 13, and 21% of non-electroporated cells, respectively.

## Discussion

### Effects of high-intensity light on the content of chlorophyll *a* and *b* in *E. gracilis* cells

We previously reported that continuous illumination at an intensity of ~460 μmol m^−2^ s^−1^ appears to be a threshold of light stress that can be tolerated by *E. gracilis* grown at 25 °C [32]. In our present study, although the concentration of cells in cultures grown under illumination at 240 μmol m^−2^ s^−1^ was similar to that of control, chlorophyll content was significantly decreased in those cells after cultivation for 7 days (Table 1). This result suggests that illumination at 240 μmol m^−2^ s^−1^ can also induce light stress for this alga.

Steinbrenner and Linden [26] reported that illumination at 10–250 μmol m^−2^ s^−1^ decreases the total chlorophyll content in *H. pluvialis* cells in a light intensity-dependent manner. Similarly, Lamers et al. [29] reported that the total chlorophyll content of *D. salina* cells decreases in response to a stepwise increase in light intensity from 150 to 650 μmol m^−2^ s^−1^. These data are compatible with our findings.

### Ultrastructure of *E. gracilis* cells grown under high intensity light

We found that treatment of *E. gracilis* cells with high-intensity light (920 μmol m^−2^ s^−1^) caused an increase in cell size and fresh weight (Fig. 1 and Table 1). Light-induced cell swelling has also been observed in *D. salina*. Lamers et al. [29] found that high-intensity light caused cell division arrest and increased the volume of *D. salina* cells immediately after the shift from 200 to 1400 μmol m^−2^ s^−1^. Our TEM study revealed that cells illuminated at 920 μmol m^−2^ s^−1^ accumulated more paramylon granules than control cells (Fig. 1b and c). This result is consistent with a report of light-induced starch accumulation in *Dunaliella bardawil* cells [33]. Accumulation of paramylon granules in *E. graciilis* might be due to the light stress-induced cell division arrest and would be the cause of cell swelling.

TEM revealed that the number of thylakoid layers clearly decreased in *E. gracilis* illuminated at 920 μmol m^−2^ s^−1^ (Fig. 1d and e). This decrease coincided with a significant decrease in chlorophyll content in these cells (Table 1). Considering that the chlorophyll *a*/*b* ratio of cells remained constant under various light intensities, the decrease in the chlorophyll *a* and *b* content in the cells was likely caused by a decrease in thylakoid layers in chloroplasts (Table 1 and Fig. 1). In contrast, exposure to high-intensity light induced the accumulation and enlargement of lipid globules in chloroplasts and the cytoplasm of these cells (Fig. 1). These findings are in accordance with previous reports of light-induced formation of lipid globules in *D. salina* [29] and *D. bardawil* [33].

### Effects of high-intensity light on the relative content of carotenoids in *E. gracilis* cells

Our results revealed that the carotenoid content of *E. gracilis* increased in response to increasing light intensity (Fig. 3). This light-induced accumulation of carotenoids has been reported for several other green algae. In *C. zofingiensis*, for example, illumination at 150 μmol m^−2^ s^−1^ markedly increased the contents of zeaxanthin, canthaxanthin and astaxanthin [30]. Wang et al. [34] showed that illumination at 350 μmol m^−2^ s^−1^ induced astaxanthin accumulation and increased the total carotenoid content in *H. pluvialis*. Lamers et al. [29] reported that the β-carotene content of *D. salina* cells increased in response to an increase in light intensity from 150 to 650 μmol m^−2^ s^−1^, and they found that high-intensity light (1400 μmol m^−2^ s^−1^) led to an increase in the content of both lycopene and β-carotene.

Unlike most flagellated green algae, the eyespot apparatus (carotenoid-rich lipid globules) of *E. gracilis* is located in the cytoplasm [35]. Heelis et al. [36] reported that the major carotenoids in the eyespot globules in this alga are β-carotene, Ddx and Dtx. In addition, Cunningham and Schiff [37] observed that *Euglena* cells contain carotenoids in extraplastidic pools. Thus, the increase in these three carotenoid species in *E. gracilis* cells in response to high-intensity light was considered to be partly due to the accumulation of cytoplasmic lipid globules in addition to the accumulation of plastoglobules (Figs. 1 and 3).

Our HPLC analyses showed that, under the high-intensity light (920 μmol m^−2^ s^−1^), the pool size of diadinoxanthin cycle pigments (Ddx + Dtx) in *E. gracilis* cells increased by 1.2-fold relative to control cells, and the Dtx/Ddx ratio increased from 0.05 (control) to 0.09 (920 μmol m^−2^ s^−1^). In diatoms, it has been reported that a larger pool of Ddx promotes non-photochemical quenching [38], and the concentration of Dtx correlates directly with non-photochemical quenching in *Phaeodactylum tricornutum* [38, 39]. The increase in the Dtx/Ddx ratio of *E. gracilis* caused by illumination at 920 μmol m^−2^ s^−1^ suggests that Dtx participates in photoprotection of this alga during the long-term acclimation to high-intensity light.

### Suppression of Eg*crtB* expression

Blocking carotenoid biosynthesis in *E. gracilis* by transient suppression of Eg*crtB* by RNAi caused chlorosis in cells and remarkably decreased the cell concentration and content of chlorophyll and carotenoid (Table 2 and Figs. 4 and 5). These results agree with previous studies showing that defects in phytoene synthase lead to a lack of or striking decrease in chlorophyll and carotenoid content in *Chlamydomonas reinhardtii* [40] and *Scenedesmus obliquus* [41]. McCarthy et al. [40] reported that the *C. reinhardtii* mutant *lts1* with a defective *PSY* gene has a very pale-green phenotype and contained much less chlorophyll than the wild-type strain. A considerable decrease in chlorophyll concentration has also been observed in the *C-6E* mutant of *S. obliquus* with a defect in phytoene synthase [41]. Similarly, the *S. obliquus* mutant *C-6E* synthesizes only trace amount of carotenoids owing to a defect in the formation or function of phytoene synthase [41].

We did not observe any obvious difference in the shape of chloroplasts and thylakoid membranes between the non-electroporated cells and Eg*crtB*-suppressed cells in the TEM study, although the number of thylakoid layers of chloroplasts was slightly decreased by treatment with Eg*crtB*-dsRNA (Fig. 4c). These results suggest that the observed significant decrease in carotenoid content in Eg*crtB*-suppressed cells (Fig. 5) was likely due to the decrease in the number or size of chloroplasts.

The content of all major carotenoid species in *E. gracilis* markedly decreased in the same way by suppression of Eg*crtB* expression (Additional file 2 and Fig. 5). This result indicates that phytoene synthesis is considered to be the first committed and rate-limiting step of the carotenoid biosynthesis also in this alga. Our TEM study indicated that Eg*crtB*-suppressed cells accumulated paramylon granules in the cytoplasm (Fig. 4c), similar to cells illuminated at 920 μmol m^−2^ s^−1^ (Fig. 1c). In addition, although the pool size of diadinoxanthin cycle pigments (Ddx + Dtx) decreased by 86% with treatment with Eg*crtB*-dsRNA compared with non-electroporated cells, the Dtx/Ddx ratio increased from 0.06 (non-electroporated) to 0.10 in Eg*crtB*-suppressed cells. As mentioned above, a significant increase in the Dtx/Ddx ratio was observed under high-intensity light (Fig. 3). Hence, these results indicate that the Eg*crtB*-suppressed cells were light-stressed under illumination, even at 55 μmol m^−2^ s^−1^ because of carotenoid depletion. McCarthy et al. [40] proposed that chlorophylls in *lts* mutants of *C. reinhardtii*, which are unable to grow in the light–even very low-intensity light–would cause photooxidative stress in cells by acting as photosensitizers in the absence of carotenoids.

## Conclusions

We found that the carotenoid content in *E. gracilis* cells increased in response to high-intensity light. Accumulation of carotenoids in these cells appeared to be associated with an increase in lipid globules in chloroplasts and the cytoplasm of this alga under the higher-intensity light conditions. Our results also revealed that suppression of Eg*crtB* resulted in a significant decrease in the content of carotenoids and led to an increase in the Dtx/Ddx ratio, as observed with the high-intensity light treatment. This study indicates that Dtx contributes to photoprotection of *E. gracilis* during long-term acclimation to light-induced stress.

## Methods

### Biological materials

*Euglena gracilis* Klebs (strain Z) was grown in 100 ml of Cramer-Myers medium [42] containing 0.1% ethanol at an initial cell concentration of 3 × 10^3^ cells ml^−1^ in a 300-ml conical flask. The cells were cultured at 25 °C under continuous illumination at 27, 55 (control), 240, 460, and 920 μmol m^−2^ s^−1^ for 7 days as we reported previously [32]. Cell number was counted daily under a microscope using a plankton counter (MPC200, Matsunami Glass, Osaka, Japan). For the analysis of expression level of Eg*crtB* and determination of the content of carotenoids and chlorophyll, algal cells were harvested by centrifugation (3000×*g*, 2 min) and stored at −60 °C until the measurement.

### Determination of chlorophyll *a* and *b* content in *E. gracilis* cells

For the determination of chlorophyll *a* and *b* in *E. gracilis*, pigments were extracted from cells three times with 1 ml of buffered aqueous 80% acetone [43]. Concentrations of chlorophyll *a* and *b* in the extracts were determined by the absorption with extinction coefficients reported by Porra et al. [43].

### Extraction of carotenoids from *E. gracilis* cells and HPLC analysis

Under dim light, pigments were extracted twice from cells with 1 ml of acetone/methanol (7:2, *v*/v) immediately before HPLC analyses. After centrifugation, extracts were dried with a rotary evaporator. The residue was dissolved in chloroform/methanol (3:1, *v*/v) and then analyzed with an HPLC system equipped with Mightysil RP-18 GP analytical column (4.6 mm × 150 mm, 5 μm particles, Kanto Chemical, Tokyo, Japan) and guard column (4.6 mm × 5 mm, 5 μm particles, Kanto Chemical, Tokyo, Japan). The elution conditions were as follows: 0–10 min, linear gradient from 90% methanol/H_2_O (*v*/v) to 100% methanol; 10–50 min, isocratic 100% methanol at 1.0 ml min^−1^. Absorbance spectra (250–700 nm, 1.2 nm resolution) and retention times were recorded with an SPD-M20A Photodiode Array Detector (Shimadzu, Kyoto, Japan).

The composition of the major carotenoids was calculated from these molar absorption coefficients and areas under the peak in the chromatogram of absorbance at 445 nm. Relative carotenoid content per cell was calculated by normalizing molar ratios of major carotenoid species to chlorophyll *a* (carotenoids/chlorophyll *a*) based on absorbance at 445 nm in HPLC analysis with chlorophyll *a* content per cell (mol cell^−1^) determined as described above.

### TEM

For our TEM study, cells were harvested by centrifugation (1000×*g*, 2 min), and fixed with 1.7% glutaraldehyde in 50 mM sodium cacodylate buffer (pH 7.0) for 2 h and then post-fixed in 2% osmium tetroxide in the same buffer for 2 h at room temperature. After dehydration in an ethanol series, fixed cells were embedded in Spurr’s resin. Ultrathin sections (80 nm thick) were cut with a diamond knife on an ULTRACUT E ultra-microtome (Leica, Wetzlar, Germany) and mounted on Formvar-coated grids. Sections were stained with 4% uranyl acetate for 18 min and 0.4% lead citrate solution for 7 min at room temperature and observed on a JEM-1400 instrument (JEOL, Tokyo, Japan) at 120 kV.

### RNAi-mediated suppression of Eg*crtB*

Expression of Eg*crtB* in *E. gracilis* was transiently silenced with dsRNA-mediated interference as described by Iseki et al. [44]. For the synthesis of the template for Eg*crtB*-dsRNA, part of the Eg*crtB* cDNA (DDBJ accession No. LC062707) was amplified (472-bp fragment) by PCR with PrimeSTAR GXL Polymerase (Takara Bio, Shiga, Japan) and the primers 5′-TAATACGACTCACTATAGGGCAGCCGTACTACGACATGA-3′ and 5′-TAATACGACTCACTATAGGGGGATCTGGCTGTAGAGGTC-3′, which contain T7 RNA polymerase promoter sequence. The dsRNA of partial Eg*crtB* was synthesized with the MEGAscript T7 Transcription kit (Thermo Fisher Scientific, Massachusetts, USA).

The Eg*crtB*-dsRNA was introduced into *E. gracilis* cells with an electroporator (Micropulser, Bio-Rad, California, USA). Specifically, 2.0 × 10^6^ cells were electroporated eight times at 0.4 kV with or without 15 μg of Eg*crtB*-dsRNA in 100 μl CM medium in a 0.2-cm gap cuvette (Bio-Rad, California, USA). Subsequently, cells were inoculated in 100 ml of CM medium containing 0.1% ethanol at an initial cell concentration of 3 × 10^3^ cells ml^−1^ and cultivated for 7 days at 25 °C under continuous light at 55 μmol m^−2^ s^−1^ with agitation (90 rpm). Cells that had not been treated with Eg*crtB*-dsRNA or electroporated were used as non-electroporated cells.

Expression of Eg*crtB* in cells with or without Eg*crtB*-dsRNA was analyzed by semi-quantitative reverse transcription-PCR. Total RNA was extracted from cells using the RNAqueous kit (Thermo Fisher Scientific, Massachusetts, USA) and Plant RNA Isolation Aid (Thermo Fisher Scientific, Massachusetts, USA). First-strand cDNA was synthesized from total RNA with the QuantiTect Reverse Transcription kit (Qiagen, Hilden, Germany) and used as the template. *GAPDH* expression was used as an internal control for the semi-quantitative RT-PCR analysis. PCR was conducted with EmeraldAmp MAX PCR Master Mix (Takara Bio, Shiga, Japan). Primer sequences were as follows: *GAPDH*, 5′-GGTCTGATGACCACCATCCAT-3′ and 5′-CGACGACACGGTTGGAGTAT-3′; Eg*crtB,* 5′-CAGCCGTACTACGACATGATC-3′ and 5′-GGATCTGGCTGTAGAGGTCC-3′.

## Additional files

Additional file 1: Figure S1. Effects of light intensity on carotenoid composition of *E. gracilis* cells. **(A–E)** HPLC chromatogram (445 nm) of extracts from *E. gracilis* grown under illumination at 27 **(A)**, 55 **(B)**, 240 **(C)**, 460 **(D)**, or 920 μmol m^−2^ s^−1^ **(E)** for 7 days. (*Insets*) Same chromatograms with an expanded *y* axis. mAU, milli-absorbance units. 1, neoxanthin; 2, diadinoxanthin; 3, all *trans*-diatoxanthin; 4–6, *cis*-diatoxanthin; 7, chlorophyll *b*; 8, chlorophyll *a*; 9, β-carotene (PDF 96 kb)

Additional file 2: Figure S2. Effects of suppressing Eg*crtB* on carotenoid composition of *E. gracilis* cells. **(A–C)** HPLC chromatogram (445 nm) of extracts from *E. gracilis* cells treated without electroporation or Eg*crtB*-dsRNA (non-electroporated) **(A)**, or cells treated with **(C)** or without Eg*crtB*-dsRNA **(B)**. (*Insets*) Same chromatograms with an expanded *y* axis. mAU, milli-absorbance units. 1, neoxanthin; 2, diadinoxanthin; 3, all *trans*-diatoxanthin; 4–6, *cis*-diatoxanthin; 7, chlorophyll *b*; 8, chlorophyll *a*; 9, β-carotene (PDF 69 kb)

## Abbreviations

CrtB, PSY: Phytoene synthase
Ddx: Diadinoxanthin
dsRNA: Double-stranded RNA
Dtx: Diatoxanthin
HPLC: High-performance liquid chromatography
RNAi: RNA interference
ROS: Reactive oxygen species
TEM: Transmission electron microscopy/microscopic
