# Supplementary material for: Suppression of the phytoene synthase gene (EgcrtB) alters carotenoid content and intracellular structure of Euglena gracilis
Source: BMC Plant Biol. 2017 Jul 17;17:125. doi: 10.1186/s12870-017-1066-7 (PMC5513367; doi:10.1186/s12870-017-1066-7)
Supplement: Supplementary file 1 — Effects of light intensity on carotenoid composition of E. gracilis cells. (A–E) HPLC chromatogram (445 nm) of extracts from E. gracilis grown under illumination at 27 (A), 55 (B), 240 (C), 460 (D), or 920 μmol m−2 s−1 (E) for 7 days. (Insets) Same chromatograms with an expanded y axis. mAU, milli-absorbance units. 1, neoxanthin; 2, diadinoxanthin; 3, all trans-diatoxanthin; 4–6, cis-diatoxanthin; 7, chlorophyll b; 8, chlorophyll a; 9, β-carotene (PDF 96 kb) [file 12870_2017_1066_MOESM1_ESM.pdf]

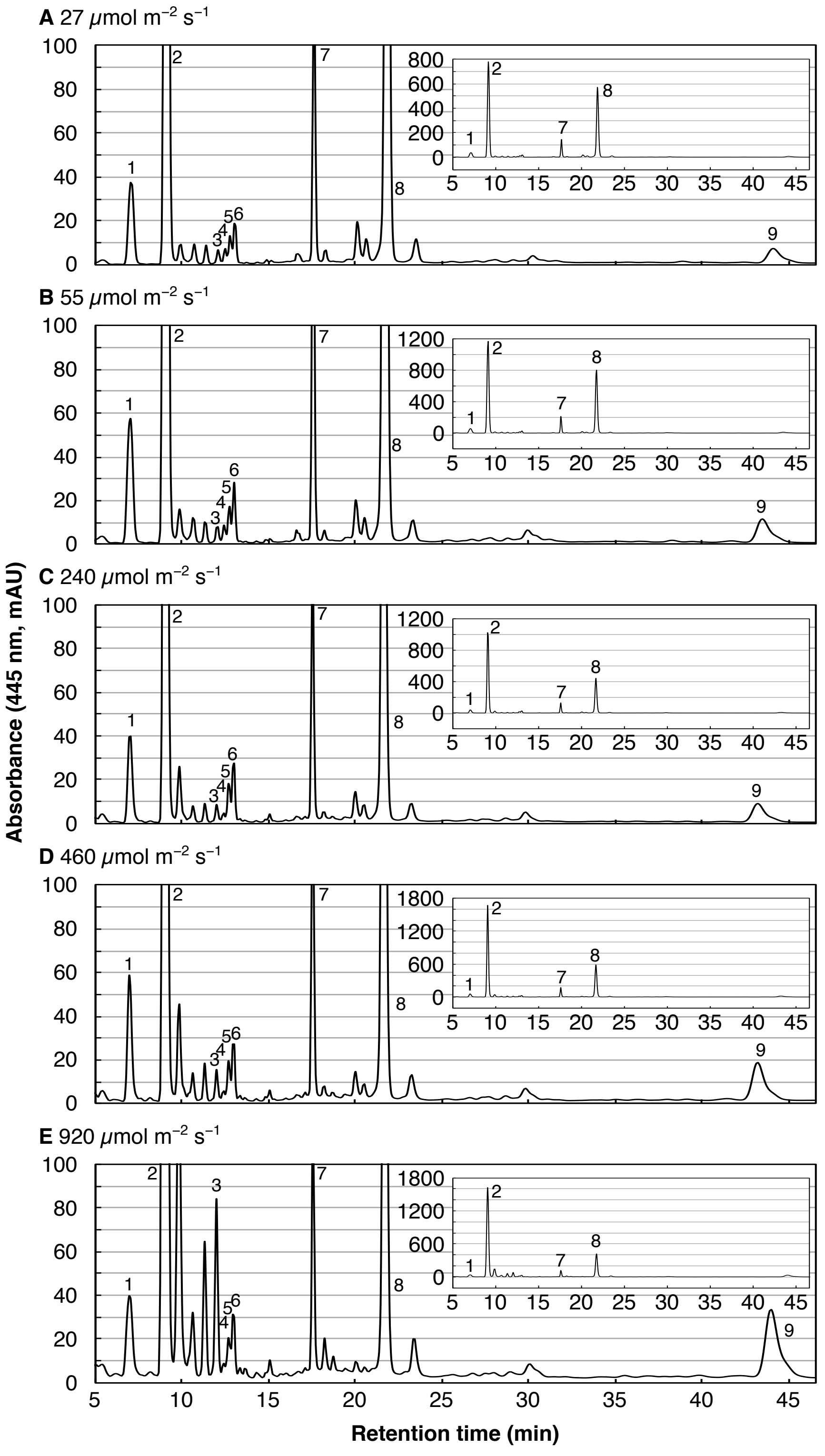
A 27 μmol m−2 s−1
B 55 μmol m−2 s−1
C 240 μmol m−2 s−1
D 460 μmol m−2 s−1
E 920 μmol m−2 s−1
Absorbance (445 nm, mAU)
Retention time (min)
1
2
3
4
5
6
7
8
9
