# Supplementary figures and images for: Suppression of the phytoene synthase gene (EgcrtB) alters carotenoid content and intracellular structure of Euglena gracilis
Source: BMC Plant Biol. 2017 Jul 17;17:125. doi: 10.1186/s12870-017-1066-7 (PMC5513367; doi:10.1186/s12870-017-1066-7)

**A** non-electroporated

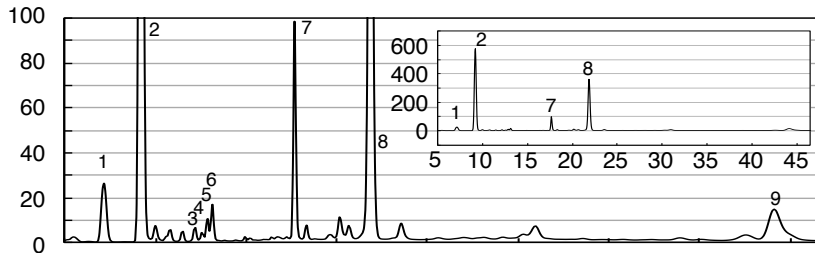

**B** *EgcrB*-dsRNA (-)

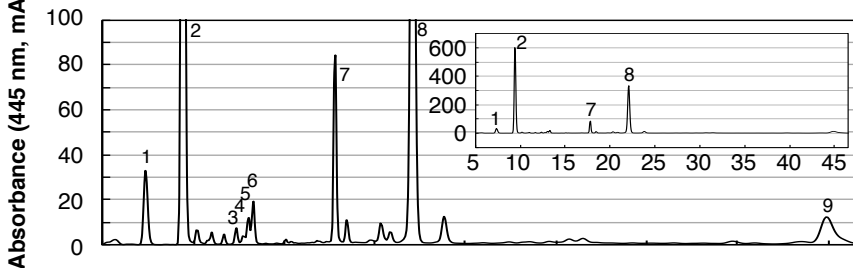

**C** *EgcrB*-dsRNA (+)

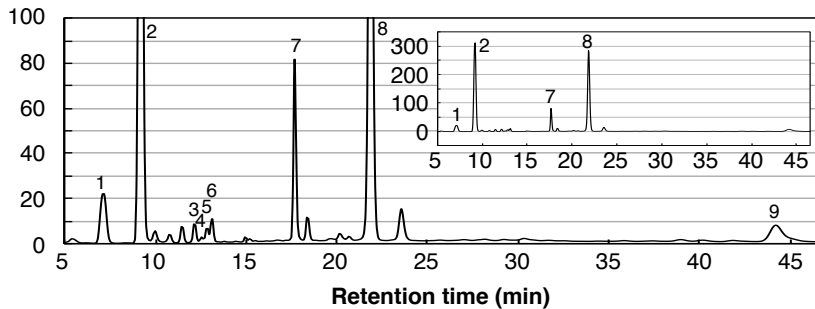

Supplement: Supplementary file 2 — Effects of suppressing EgcrtB on carotenoid composition of E. gracilis cells. (A–C) HPLC chromatogram (445 nm) of extracts from E. gracilis cells treated without electroporation or EgcrtB-dsRNA (non-electroporated) (A), or cells treated with (C) or without EgcrtB-dsRNA (B). (Insets) Same chromatograms with an expanded y axis. mAU, milli-absorbance units. 1, neoxanthin; 2, diadinoxanthin; 3, all trans-diatoxanthin; 4–6, cis-diatoxanthin; 7, chlorophyll b; 8, chlorophyll a; 9, β-carotene (PDF 69 kb) [file 12870_2017_1066_MOESM2_ESM.pdf]
